# Wrist-Based Fall Detection: Towards Generalization across Datasets

**DOI:** 10.3390/s24051679

**Published:** 2024-03-05

**Authors:** Vanilson Fula, Plinio Moreno

**Affiliations:** 1 Instituto Superior Técnico, Universidade de Lisboa, 1049-001 Lisboa, Portugal; vanilsonfula@tecnico.ulisboa.pt; 2 Institute for Systems and Robotics, LARSyS, Torre Norte Piso 7, Av. Rovisco Pais 1, 1049-001 Lisboa, Portugal

**Keywords:** fall detection system, wrist devices, false alarm, unbalanced learning, sliding window

## Abstract

Increasing age is related to a decrease in independence of movement and with this decrease comes falls, millions of falls occur every year and the most affected people are the older adults. These falls usually have a big impact on health and independence of the older adults, as well as financial impact on the health systems. Thus, many studies have developed fall detectors from several types of sensors. Previous studies related to the creation of fall detection systems models use only one dataset that usually has a small number of samples. Training and testing machine learning models in this small scope: (i) yield overoptimistic classification rates, (ii) do not generalize to real-life situations and (iii) have very high rate of false positives. Given this, the proposal of this research work is the creation of a new dataset that encompasses data from three different datasets, with more than 1300 fall samples and 28 K negative samples. Our new dataset includes a standard way of adding samples, which allow the future addition of other data sources. We evaluate our dataset by using classic cost-sensitive Machine Leaning methods that deal with class imbalance. For the training and validation of this model, a set of temporal and frequency features were extracted from the raw data of an accelerometer and a gyroscope using a sliding window of 2 s with an overlap of 50%. We study the generalization properties of each dataset, by testing on the other datasets and also the performance of our new dataset. The model showed a good ability to distinguish between activities of daily living and falls, achieving a recall of 90.57%, a specificity of 96.91% and an Area Under the Receiver Operating Characteristic curve (AUC-ROC) value of 98.85% against the combination of three datasets.

## 1. Introduction

Aging is a natural human process, which is accompanied by psychological, physical and social changes, causing people to lose some of their independence. With the evolution of technology and the most different therapeutic methods that are emerging, people have a life expectancy of at least 60 years. According to the WHO between 2015 and 2050, the proportion of the world’s population over the age of 60 will almost double from 12% to 22%. In 2020, the number of people aged 60 and over outnumbered children under 5 years of age. By 2050, the number of people aged 60 and over will double (2.1 billion) [1]. Each year, an estimated 684,000 individuals die from falls worldwide. Adults over 60 years of age suffer the highest number of fatal falls [2]. According to [3], high prevalence rates of Fear of Falling were found on older adults people who have already experienced a fall, due to the experience, develop a fear of falling again, a fear that ends up causing greater difficulty in the level of mobility and reducing the independence of the older adults, thus making them more susceptible to future falls. Falls are defined as an event that results in a person inadvertently coming to rest on the ground or floor or a lower surface [2]. However, there are different ways of assessing falls and their related injuries, which hampers the injury incidence and mortality rates. The burden of falls on the healthcare systems and the prevention plans is very critical [4]. Fall-related injuries can be fatal or non-fatal, although most are not fatal and to a large extent predictable. Older adults have tried fall detectors in various pilot studies, where the subjects felt more secure by using the devices [5,6,7] but the main concern was the large amount of false positives [8].

The fact that falls can result in accidents that may require intensive treatment and in the worst cases lead to death, many studies on Fall Detection Systems (FDS) have been developed. However, due to the risks and costs associated with acquiring data for FDS, most falls-related datasets have a limited number of participants, this can result in small and therefore unrepresentative datasets. In response to these challenges, innovative approaches are sought, such as combining datasets from different sources, to overcome limitations associated with data acquisition for FDS.

It is important to note that in real life and in all datasets, there is a natural imbalance in data, especially when we consider how often falls occur. Recognizing the propensity of Machine Learning (ML) algorithms to develop biased models in situations of data scarcity, this work aims to overcome this limitation by considering multiple datasets recorded in different contexts. The strategy involves diversifying fall scenarios, ensuring that the common denominator is the use of wearables devices on the wrist to measure accelerations and wrist pose relative to the inertial reference frame. This approach aims to build fall detection models that are more robust and representative of reality, considering the challenges posed by unbalanced learning.

## 2. Related Work

### 2.1. Classification Models in ML

ML develops classification and regression models based on the dataset and data trends. ML models can be categorized into three main types: supervised, unsupervised, and reinforcement learning. There are several ML algorithms widely used in previous FDS, including Support Vector Machines SVM ([9,10]), Artificial Neural Networks ANN ([10]), k-Nearest NeighborskNN ([9,10,11]), Decision Trees DT ([9]) and Random Forest RF ([10]). Let us introduce the notation of our problem. The samples of the training set:

(1) $$T=\left(x^{\left(1\right)},y^{\left(1\right)}\right),\left(x^{\left(2\right)},y^{\left(2\right)}\right),\ldots ,\left(x^{\left(n\right)},y^{\left(n\right)}\right),$$

where *n* is the number of examples in the dataset, $x^{\left(i\right)}$ is the feature vector of the *i*th example, and $y^{\left(i\right)}$ is the label associated with this example. Our model aims to estimate the classification function:

(2) $$\hat{y}=f_{\theta }\left(x\right),$$

where *f* corresponds to the classification function with parameters θ, that estimates $\hat{y}$ as close as possible to the actual value *y*. To estimate the parameters θ of the function *f*, a loss function is defined (3) for SVM and (4) for ANN:

(3) $$argmin_{w,b,\xi }\frac{1}{2}{\left||w|\right|}^{2}+C\sum_{i=1}^{n}\left(\omega_{y^{\left(i\right)}}\xi_{i}\right),$$

(4) $$J\left(W,\theta \right)=\frac{1}{2n}\sum_{i=1}^{n}\omega_{y^{\left(i\right)}}{\left({\hat{y}}^{\left(i\right)}-y^{\left(i\right)}\right)}^{2},$$

where $\omega_{y^{\left(i\right)}}$ represents the weights of the training data contributions based on the data classes, allowing the model to give more importance to the minority class. In the case of SVM (3), *w* represents the vector of weights, which is used to define the separating hyperplane between classes in a feature space, the variable $\xi_{i}$ is known as the slack variable, *C* is called the parameter cost and represents the slack penalty. In the case of ANN (4), $W=(W_{1},W_{2},\ldots ,W_{n})$ represents the synaptic weights between the neurons in the network, $\theta =(\theta_{1},\theta_{2},\ldots ,\theta_{n})$ represents the model parameters that are tuned during training and J(θ) is the cost function that measures how well the Model performance fits the training data. The Equation (4) represents the mean squared error.

#### Imbalanced Learning

Standard ML models face challenges when dealing with imbalanced datasets, resulting in metrics biased towards the majority class. To overcome this problem, new principles and tools are needed that can efficiently transform large volumes of unbalanced data into representative knowledge [12]. Fall detection faces challenges due to the rarity of fall events, resulting in insufficient training data. Standard supervised learning methods may have some problems dealing with insufficient data [13]. In [14] a taxonomy was proposed considering the availability of falls data, identifying classification methods based on this data availability. Two main approaches for dealing with imbalanced datasets are sampling methods [15,16], adjusting the distribution of classes, and cost-sensitive methods, assigning different costs to classifications [17,18]. In [19] it discusses classification algorithms for dealing with imbalanced datasets. The first is the use of ensemble methods, which combine several independent classifiers to improve classification robustness, including techniques such as bagging and boosting. The second approach involves modifications to classification algorithms to improve their ability to learn from unbalanced sets, such as assigning different weights to classes or introducing different costs for classification errors in majority and minority classes. Finally, some of the evaluation metrics that work for balanced datasets, provide biased results (e.g., accuracy, using only Precision) towards the majority class. Nevertheless, others work well, such as Area Under the Curve(AUC), G Mean, F Measure [19].

Our aim is to use as much data as possible, so we follow the cost sensitive approach, setting $\omega_{y^{\left(i\right)}}$ in (3) and in (4), according to the class frequency. In addition, we use metrics such as Area Under the Curve (AUC), F Measure, as well as senstivity (i.e., recall) and specificity.

### 2.2. Types of FDS

Systems related to falls follow two main lines of research: Fall detection and fall prevention [20]. The main objective of fall detection is to accurately detect whenever a fall occurs [21], fall prevention aims to predict the fall through gait and body balance analysis to recognize people at higher risk of falling [22]. Of the various FDS that are emerging, we can classify the FDS into: camera-based, using context/ambient sensors and wearables [23].

#### 2.2.1. Camera-Based FDS

Cameras are installed in the premises of a specific area to continuously monitor the activities of older adults people, and the fall incident is detected based on image or video processing techniques [23]. In [24] a camera-based FDS for smart homes was developed, the results using the kNN classifier showed a detection rate greater than 96% on more than 50 different fall videos.

#### 2.2.2. Context/Ambient FDS

Generally, optical sensors, audio sensors, pressure sensors, infrared sensors, and other ambient sensors are combined and deployed in the environment to detect falls [25,26]. In [27] a real-time context-aware FDS was proposed for the older adults, which used a sensor positioned under a carpet to track walking activity for fall monitoring.

#### 2.2.3. Wearables FDS

Signals are captured by motion sensors that can detect a fall, a sensing device is placed at a position on the body to collect data. These systems have the advantage of being portable and can be used anywhere, they can be used indoors and outdoors, without being limited to a specific place for fall detection. The wearables FDS are easy to install, economical and in many cases do not violate privacy restrictions, thus being the best option to support elderly. In [28] was demonstrated that the position of the wearables sensor is crucial for the effectiveness of FDS. In [29] a study was conducted on how the position of the wearables sensor affects fall detection sensitivity, the sensors were positioned on the head, chest, waist, right wrist, right thigh and right ankle. The waist region stood out with 99.96% fall detection sensitivity using the kNN while the best sensitivity achieved by the wrist sensor is 97.37%, although this location is highly preferred for current wearables applications due to their convenience and removing medical stigma.

In [28] research was carried out on a smartphone-based architecture with multiple mobility sensors wearables for fall detection, using fall detection algorithms based on threshold methods. The sensors were placed on the chest, waist, left wrist, right ankle and right thigh to evaluate the ability to detect falls, the results showed that the wearables sensor located on the wrist had the best ability to detect falls. In [11], a work was presented on a fall detector using an accelerometer coupled to a wearables device located on the wrist, a 3NN algorithm is trained. To evaluate the solution, volunteers were asked to wear the watch with the model for the entire life of the watch’s battery, they were encouraged to play with the device as they pleased, try to induce false positives or simply live a normal day. Older volunteers were not encouraged to perform any falls, and only 25 false positives and 3 false negatives were noted in seven tests carried out on five individuals over 238 h, or almost ten full days. During the period, 11 of the 14 falls were identified. In [10] the UP-Fall dataset was used to identify falls using ML methods such as RF, SVM, MLP and kNN. Using RF an accuracy of 95.76% was achieved by combining inertial sensors.

### 2.3. Datasets

The creation of a dataset for FDS is done in a controlled environment, where the actions to be monitored are carried out intentionally. The Table 1 lists some datasets, which are distinguished in several ways. Most of the proposed datasets have a large amount of ADL information and do not have sufficient information about fall varieties, and the performance of FDS is affected by the lack of diversity and the number of samples in each class.

According to [14], storing real-life datasets of falls of older adults often requires complex setups, long-term facilities, cooperation from external institutions, ethical permissions, large time investments, high operating costs, and beyond. Furthermore, they also require a high level of computational resources for analysis, as well as a long process of recording, extracting and labeling data. Due to these difficulties in obtaining data on real falls, most studies on FDS have been carried out using simulated data sets, meaning that falls do not occur naturally. Although these datasets are created in controlled environments, however obtaining simulated data for FDS is also challenging due to the risks associated with simulating falls and the costs of data acquisition, which include specialized equipment, human supervision, and the large investment of time. Normally the volunteers are healthy young people and in a few cases, when there are elderly people, they only simulate ADL due to the risks associated with falls.

### 2.4. Wrist-Based FDS

The mismatch between multiple datasets for training and testing results in FDS with high accuracy during validation with the same dataset. However, this accuracy is not maintained when a different data set is used to validate these FDS. In the study by [9], a solution for detecting falls using a wrist device was developed. The work compared threshold-based and ML methods, involving 22 participants in 12 activities. The threshold-based method achieved 91.1% accuracy, while ML approaches, especially SVM, surpassed this accuracy, highlighting the effectiveness of ML in detecting falls compared to threshold-based methods. The study has limitations when using a single dataset for training and validation, leading to optimistic results that may not reflect real situations. Furthermore, it does not consider the impact of data imbalance on the results obtained.

In [38], three datasets were combined for an FDS, and it was noticed that the performance of the FDS is affected when different datasets are used for evaluation and training. It was also seen that the datasets have different generalization capabilities depending on the variability of movements that each one presents, while ML algorithms seem less sensitive to sampling frequency or acceleration interval. In [39] the authors analized the challenges faced by FDS using wrist-worn devices, with four datasets (UMA Fall, TST, DaLiaC, and UNIOVI Simulated Epilepsy) and using ML techniques. UMA Fall was defined for training and testing, while the other three were used for validation. The results showed that the models do not provide sufficient generalization capabilities and that it is necessary to establish a taxonomy for falls so that the data sets all have the same fall dynamics.

In [40], a Convolutional Neural Network was applied to an FDS using 14 sets of data collected by an accelerometer. The initial evaluation with the SisFall dataset outperformed previous investigations, but when training and testing with 13 other datasets, performance decreased significantly. The influence of the training data set on the model’s performance stands out, attributing this to the variability of movements between the sets. In [41], an FDS was developed using three sets of data from a wrist sensor, resulting in a fall detection accuracy higher than previous studies with the same sets. The robustness of the model was verified through validations between sets and within each set, highlighting the most influential features for detection. The model achieved high accuracy in detecting falls, demonstrating the effectiveness of wrist devices. With an accuracy of 78.51 + 4.06%, the diversity and similarity between the types of falls and ADL impacted the validation of the model. The studies in [38,39,40,41] are similar to this work, being limited by the absence of training methods that combine data sets to increase the variability of movements and for not considering the influence of data imbalance on the results.

## 3. Selection of Datasets

### 3.1. Datasets

Acquiring datasets for training fall detection algorithms is challenging due to risks, time and resource limitations, resulting in generally small datasets. This compromises the variability of fall samples and affects the generalization capacity of the model, generating uncertainty about its effectiveness. This work seeks to understand the realistic limits of fall detection by analyzing diverse datasets that represent different acquisition conditions and contexts. The main focus is on sets in which information about falls and ADL are recorded by wrist devices with accelerometers and gyroscopes. The proposed model highlights the use of these devices, considered advantageous, practical and sensitive to body movements for detecting falls, integrating low energy consumption sensors.

Datasets in which information was collected by devices positioned on the wrist stand out, including UMA FALL, UP FALL, FallAllD, DU-MD [42], CMDFALL [43], Smartwatch, WEDA FALL, TST Fall Detection [44] and Gravity Project [45]. The DU-MD, CMDFALL, Smartwatch, TST Fall Detection and Gravity Project datasets exclusively consider the accelerometer for data extraction. Among these sets, those aligned to this project based on the sensors of interest (accelerometer and gyroscope) are only UMA Fall, UP Fall, WEDA Fall and FallAllD. It is worth mentioning that the FallAllD dataset will not be included in this work due to the distinct categories of falls and ADL, which differ significantly from the other three datasets aligned to this work.

#### 3.1.1. Dataset Up Fall

UP Fall is a multimodal dataset that uses 14 sensors, including cameras, Kinect, accelerometers and microphones, to collect data on falls and ADL. Inertial signals were obtained from different parts of the body, ankle, neck, thigh (pocket), waist and left wrist. Table 2 lists the 11 activities, including five falls, 17 young, healthy individuals participated in three attempts per activity. Data was sampled at 18 Hz.

#### 3.1.2. Dataset WEDA Fall

The WEDA Fall dataset was collected via the Fitbit Sense wrist device, recording accelerometer and gyroscope data. With the participation of 25 individuals, including 14 young people and 11 elderly people over 80 years old, the dataset covers 8 falls and 11 ADL, as shown in Table 3. Each activity was performed in at least 3 attempts, some in 4 attempts. It should be noted that elderly participants were not asked to perform falling activities that could pose risks to their safety. The sampling rate of the dataset was 50 Hz, and its objective is to overcome the limitations of previous datasets for fall detection.

#### 3.1.3. Dataset UMA Fall

**Figure 1:**
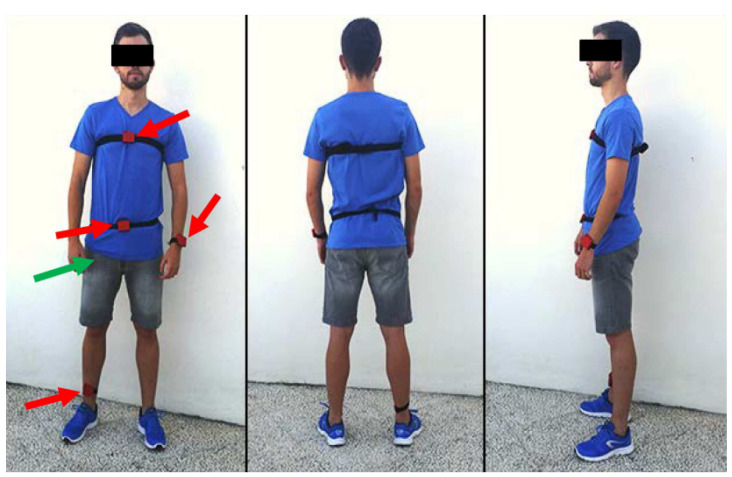
Distribution of sensors for the acquisition of the UMA Fall dataset, extracted from [36] The red arrows indicate the location of the wearable devices and the green arrow the location of the mobile phone.

The UMA Fall dataset is multimodal, using sensors in five different positions, including four wearable devices and a smartphone. The wearables were placed on the waist, chest, wrist and ankles, while the smartphone was positioned in the pants pocket as shown in Figure 1. Data were collected from 19 participants aged between 18 and 55 years, while performing ADL and simulated falls. Comprising 12 ADL and 3 falls as listed in Table 4, each activity was repeated at least three times per individual, reaching up to 18 attempts when possible. The sampling rate was fixed at 200 Hz for smartphones and 20 Hz for wearable devices, with movement monitoring for 15 s.

### 3.2. Preprocessing the Datasets

The chosen datasets come from several research institutions, preprocessing the data is crucial to standardize the format. In the UP Fall and UMA Fall sets, which are multimodal and use multiple sensors in different positions, careful selection of data from the sensors of interest is required. Given the nature of these datasets, it is essential to process each set individually to ensure uniformity and applicability of the algorithms in an analysis environment that incorporates the particularities of all chosen sets. File names follow the format established by the UP Fall dataset, which takes the form “Dataset”_SubjectXX_ActivityXX_TrialXX. Here, SubjectXX represents the subject number, ActivityXX represents the activity number, and TrialXX represents the trial number. The files were organized by data set, subjects, activities and trials. CSV files containing crucial information about participants, activities, attempts and categories (“0” for ADL and “1” for falls), coming from sensors such as gyroscopes and accelerometers, recorded by temporal marks.

The focus of this work is a binary classification, distinguishing only between falls and ADL, without considering specific types. To efficiently integrate the UMA Fall, WEDA Fall and UP Fall sets, the measurements of acceleration in *g* and angular velocity in rad/s were unified. The UMA Fall and WEDA Fall were subsampled to 18 Hz to align the frequencies with the frequency of the UP Fall dataset which is the lowest among the three sets. The UP Fall, UMA Fall, and WEDA Fall sets were manually labeled for ADL and falls at every sampled window of the time series, adjusting for differences in labeling practices between different institutions.

**Figure 2:**
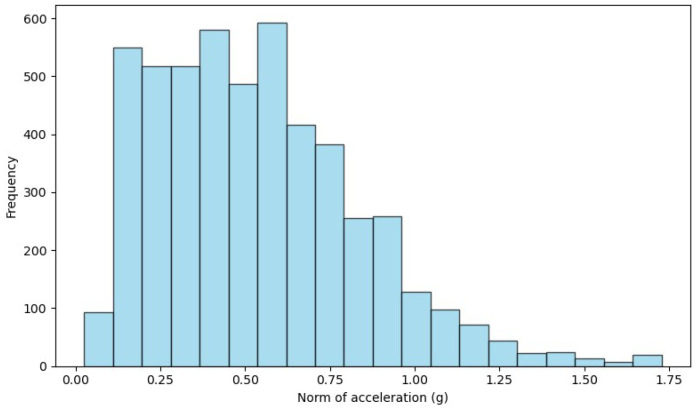
Acceleration norm histogram for the UMA Fall dataset.

**Figure 3:**
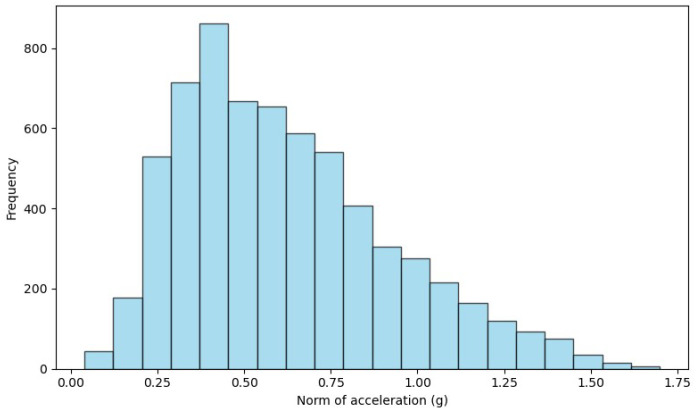
Acceleration norm histogram for the UP Fall dataset.

**Figure 4:**
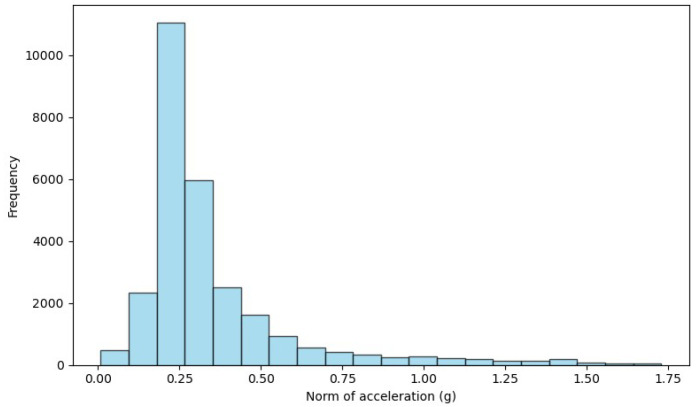
Acceleration norm histogram for the WEDA Fall dataset.

The variation in accelerometer and gyroscope scales between datasets highlights the need for preprocessing to harmonize these differences. To overcome this disparity, we opted to normalize the data, establishing a common range of [−1, 1] for accelerometers and gyroscopes. Normalization ensures that the model is trained and tested fairly, accounting for variability between datasets. This approach homogenizes the scales, makes the samples comparable and reinforces the robustness and generalization of the model in different contexts. Figure 2, Figure 3 and Figure 4 show the histrogram of the acceleration norm for each dataset. After data normalization, CSV files containing “nan” entries were removed, ensuring the quality and integrity of the dataset. Analysis of the distribution reveals a considerable imbalance, with ADL representing 95.54% and falls of only 4.46% of the total samples, characterizing an unbalanced classification problem. Table 5 shows the data final distribution, after preprocessing.

## 4. Results

**Figure 5:**
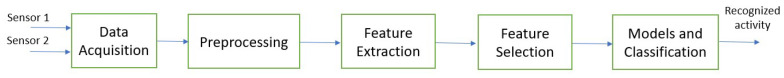
ARC methodology implemented.

Developing an effective FDS requires implementing an ARC. As Figure 5 shows, ARC integrates techniques that involve data acquisition through sensors, processing, extraction and selection of features, in addition to ML techniques, to attribute specific behaviors to the activity recognition system [46].

The input to ARC consists of data streams from sensors such as the accelerometer and gyroscope of a wrist-worn device. Preprocessing seeks to eliminate signal variability and standardize datasets to ensure uniformity. The following process involves extracting features from the input signal by segmenting the data into windows. This segmentation is crucial to capturing relevant temporal patterns in monitored activities. The extracted features, along with the labels for each class, are then used to train a classification model, and performance metrics are calculated based on the model’s predictions. This complete ARC cycle, from data entry to model evaluation, constitutes the essence of the fall detection process. Comparing classification results on this type of architectures is not easy, due to the large amount of sensing options and the cumbersome task of labeling data in a per sample basis in a consistent manner. In addition, most of the datasets consider repetitions by the same subject, which adds an extra verification step that guarantees that data from the same subject is not present on both the training and testing sets. Thus, a standardize comparison must fulfill all these requirements. We present an approach to do this based on: (i) sampling all datasets at the same rate, (ii) specifying the subject id to facilitate dataset partition construction and (iii) provide a standard manner to label the samples for fall detection, by sampling windows with 50% of overlap and labeling each one of them.

### 4.1. Feature Extraction

Feature extraction is crucial for understanding patterns in data captured by sensors in human activity recognition systems. Table 6 shows the list of the six sensor signals considered. From the sensor signals, we compute the 13 features listed in Table 7.

The feature extraction process was performed with a sliding window of 2 s and 50% overlap over the sensor feature datasets. This approach divides the time series into smaller segments, calculating specific features for each window. 50% overlap between windows ensures smooth coverage of temporal data, collecting important information over time [10]. This technique is essential for the comprehensive analysis of monitored activities, contributing significantly to training and subsequent classification. Assigning labels to the generated windows was essential, and for this, the majority criterion was used. This criterion determines that the class assigned to a window is the most frequent one within that specific window. During feature extraction with sliding windows, each temporal segment is analyzed individually, and the predominant class is determined by the mode criterion, allowing the creation of a feature dataset with labels associated with each sampled window.

### 4.2. Feature Selection

Due to storage and processing constraints on wrist devices, optimizing the efficiency of ML models becomes crucial. Feature selection is a fundamental strategy for simplifying and improving these models. Analysis of the importance of features reveals which ones play a significant role in the model. This allows you to create efficient models, ensuring high accuracy even with minimal datasets. In this way, the model adapts to the resource limitations of wrist devices, maintaining high performance in detecting activities.

The feature selection was conducted using an RF model [47]. Initially, an RF model was trained with all the characteristics of the dataset, determining the importance of each characteristic based on the Gini index [48]. Then, a second RF model was used to evaluate the contribution of each important feature to the overall accuracy, excluding one feature at a time. This approach provided information about which characteristics significantly impacted accuracy, allowing the choice of the most relevant ones for creating the detection model. The final selection included only the most important characteristics based on the Gini index that significantly contributed to improving accuracy, aiming to maximize the model’s predictive capacity and, consequently, increase its efficiency.

### 4.3. Fall Detection Models

We are facing an unbalanced classification problem, the number of samples from the negative class is significantly greater than the number of samples from the positive class. In this scenario, the effectiveness of classical ML models is compromised, as they tend to favor the majority class. To deal with this imbalance, the proposed model will adopt a specific approach to detecting falls, considering the imbalance between classes. Two common approaches in cases of imbalanced datasets are sampling methods and class cost-sensitive methods. The first involves techniques such as oversampling or undersampling. The second approach assigns different weights to classes, giving more importance to the minority class during training. The use of sampling techniques such as oversampling can introduce uncertainty into the reliability of the system, creating false fall data that can distort the real representation of falls. On the other hand, undersampling can significantly reduce ADL samples by reducing the variability and diversity of these activities in the dataset, compromising the robustness of the model to deal with the complexity of ADL in real-world situations.

Given the challenges presented, we will opt for an approach that assigns different weights to classes during training. This strategy seeks to balance the relevance of classes, giving greater importance to the minority class, without resorting to creating false data or compromising the representativeness of ADL. The attribution of weights aims to maximize the reliability and effectiveness of the FDS, seeking a precise relationship between fall detection and ADL variability. For both SVM and ANN the weight $\omega_{y^{\left(i\right)}}=n_{y^{\left(i\right)}}/n$ corresponds to the inverse class frequency, where $n_{y^{\left(i\right)}}$ represents the number of samples of class $y^{\left(i\right)}$.

For all of the models developed in this work, we use a k-fold cross validation approach (k=10) [49], ensuring that activities performed by the same person are not simultaneously in the training and testing sets. Given the imbalance between classes, model evaluation is performed using metrics that offer a more detailed and balanced view of performance. Metrics such as Recall, Specificity, AUC-ROC and F1-Score are more informative at the expense of accuracy in imbalance scenarios. To evaluate our model, the following approaches were used:

1. Train and test each dataset individually. All the datasets were labeled in a per-window method, which is the same approach on the UP-FALL dataset and explained in Section 3.2. However, we cleaned up not valide data samples and relabeled all datasets to standardize. Thus, comparisons with previous approaches serve only as reference. We do the feature selection step on each one of these datasets. Starting from 78 features, we select 27 features of UMA Fall, 28 features of UP Fall, and 28 of WEDA Fall.
2. Train and test the model with all datasets combined. The combination of approaches allows a comprehensive assessment of the performance and robustness of the proposed model in different scenarios. We also do the feature selection step on the combined dataset, which results in 26 out of 78 features.
3. Study of the generalization of the model. In this group of experiments, we train a model using samples of one dataset only, and test on the remaining datasets.

### 4.4. Model Evaluation

Before evaluating the performance of the proposed model, it is essential to highlight the performance of the base models used to test the selected datasets. This comparative analysis will serve as a reference to understand the distinctions and limitations of the proposed model in relation to the base models of each dataset.

In the study by Martinez et al. [10], classic ML algorithms were used to create a fall detection model, following the *k*-fold cross validation approach (k=10). Our model and the model in [10] follow the same overlapping window approach for sample selection, as well as the feature selection methodology. The main differences between their approach and ours, include: (i) They do not consider the imbalanced nature of the problem during training, (ii) they do not evaluate the classification result of the wrist-only wearable. Ref. [10] focuses on using multiple sensors, being the closest setup to ours the all-IMU features (i.e., wearables in several body parts). Table 8 shows the results of using only the wrist sensor for our model. On the one hand, the ANN model by Martinez et al. [10] significantly outperformed the proposed model in detecting falls, with emphasis on accuracy and specificity. Although it has a low rate of false positives and high specificity, the model had a relatively low recall, revealing a weakness in identifying falls, due to the fact that imbalance was not considered on the model optimization. On the other hand, our SVM approach has an accuracy very similar to [10], noticing that our model has a larger recall rate while keeping the specificity at high levels. It is worth noting that Martinez’s results were obtained considering the comprehensive use of sensors in five positions on the body, contributing to a more comprehensive and reliable performance on the UP Fall dataset.

Marques et al. [11] used kNN (k=3) wrist accelerometer data to extract WEDA Fall features sampled at 50Hz, following the *k*-fold cross validation approach (k=5). The main differences between our model and [11] are the raw data input and the sampling frequency. Table 9 shows that our model (SVM and ANN), using accelerometer and gyroscope data to extract WEDA Fall features and subsampling them at 18Hz, achieves a solid performance, overcoming the difference in sampling rate. The ANN showed high accuracy, robust specificity and recall, excelling in distinguishing classes and minimizing false positives and negatives in the WEDA Fall dataset.

Casilari et al. in [28] presented a real-time approach for fall detection, based on a threshold of the acceleration magnitude. The authors do not mention a cross validation approach. In addition, the labels are per recorded sequence so there are no per-window labels. However, the main similarity to our approach is the utilization of wrist-only device. Table 10 shows the results of the only the wrist-worn sensor. Although follow-up studies on UMA Fall have been published, such as [50], we show the original result published with the dataset. Metrics such as AUC and $\sqrt{S_{e}S_{p}}$ are informative indicators for unbalanced data sets, seeking balance in the identification of both classes, $S_{e}$ is recall and $S_{p}$ is specificity. The results in Table 10 indicate that for the UMA Fall dataset, the use of classical ML algorithms provides superior performance compared to threshold-based methods. Algorithms such as SVM and ANN demonstrate effectiveness in differentiating classes, exploring complex patterns in the data, including non-linear relationships, a crucial aspect in detecting falls. In contrast, threshold-based methods rely on simple rules, comparing sensor values to predefined thresholds. This simplified approach may not capture the complexity of movement patterns associated with falls, resulting in poorer performance. Regarding the position of the sensors, the results do not reveal significant differences, since in both cases the sensors are located on the wrist. Our models have similar performances to the original datasets’ models. However, our time-windows approach to labeling is a more realistic approach and challenging problem.

#### 4.4.1. Train and Test Each Dataset Individually

The model was evaluated separately for each dataset, both with normalized and non-normalized data. Normalized data did not bring improvements to the models trained on each dataset, so in Table 11 we present the results without normalization. Considering AUC-ROC and F1-Score, ANN models perform better than SVM for UMA fall and WEDA fall, while SVM performs better for UP fall. In addition, very high AUC-ROC are observed for UMA fall and WEDA fall, which is an issue of these small datasets. An important contribution of this work is to provide with a more realistic values of the metrics.

#### 4.4.2. Train and Test the Model with All Datasets Combined

Combining the datasets (UMA Fall, WEDA Fall and UP Fall), data normalization impacted the SVM and ANN algorithms. In SVM, there was an improvement in Recall, but with a reduction in other metrics, resulting in lower overall performance. ANN maintained Recall with a reduction in other metrics, affecting overall performance. Normalization increases the false positive rate, which diminish the model’s ability to distinguish falls and ADL. ANN performs better than SVM, following the same trend from the individual datasets. We observe that maximum F1-Score is around 70%, which is significantly lower than the values reported for UMA fall and WEDA fall. Thus, false positive rate is very large to apply the algorithms in realistic scenarios in Table 12.

#### 4.4.3. Study of Model Generalization

Training and testing the model on different datasets allows you to evaluate its generalization capacity and performance in adverse situations, essential for a realistic application.

Table 13 and Table 14 reveal that the models face challenges in generalizing falls, showing notable rates of false positives and false negatives. However, for ADL identification, the models show acceptable generalization capacity, with a reduced false negative rate. Data normalization does not significantly impact the overall performance of the model. In comparison mode we can say that:

1. The WEDA Fall dataset stands out in the generalization of ADL, presenting high identification capacity both with and without normalization. In one notable exception, when trained by the ANN algorithm, the UMA Fall dataset demonstrates a slight advantage, revealing better generalization ability for ADL.
2. The UP Fall dataset demonstrates the best generalization ability to fall activities, showing relatively high detection rates. In contrast, the UMA Fall dataset stands out in generalizing falls when used by the ANN algorithm.
3. The overall performance of the model reveals that the algorithms trained with the UMA Fall dataset achieved a higher AUC-ROC compared to the other datasets, indicating a greater ability to differentiate classes. This consistency reflects the quality of the characteristics or patterns present in the data. However, a notable exception occurs in the case of the SVM algorithm with data normalization, where the UP Fall set presents a superior overall performance

The results highlight the critical importance of algorithm choice and preprocessing process for model performance. Despite the flexibility of the structure, the values obtained for F1-Score and AUC-ROC indicate that the performance of the current model does not fully meet realistic scenarios. It is noted that without normalization, the data is compatible for use together, especially when sub-sampled at the lowest sampling rate between the data sets. The current model structure demonstrates flexibility and adaptability, facilitating the integration of new data sets. This expandability adds more diversity and representation to the training set and improves the generalization ability of the model. The main limitations of the combined dataset include: (i) The low frequency of the samples and the (ii) overlapping of the time-windows for the sample creation.

### 4.5. Discussion

We introduced and implemented an approach that allows to gather samples from several datasets for fall detection. We assume that the main sensor is a wearable device on the wrist of the user, which easier to put on and does not have the stigma of a medical device [11]. This assumption comes with some drawbacks that include the less accurate fall detection when compared to a similar device on the chest or waist, leading to misclassifying some ADLs as falls. We also assume that the sampling frequency of the device is 18 Hz, which allow us to include a wider amount of datasets. However, the reduced frequency also has a small reduction on the performance [11].

Our method to standardize the fall datasets differs from each one of the considered datasets, namely: (i) We use only one wrist device, (ii) our sensors include accelerometer and gyroscope, (iii) our labels are defined in a per-window manner. The previous datasets do not hold all these conditions, so we are not able to compare the classification results from previous works due to our assumptions. Nevertheless, we compared the results from previous works that matches as much as possible our standardization methdology in order to have an idea of the impact of our assumptions. Our goal is to include more datasets following our procedure, to have the largest fall dataset for wrist devices that helps to build better classification models.

Finally, our choice of classifiers was done on previous research approaches for fall detection, considering the most utilized machinle learning algorithms [21,51]. In addition, both SVM and ANN offer the possiblity of conducting class-sensitive learning, which is crucial for the fall detection datasets that have imbalanced data samples across classes.

## 5. Conclusions and Future Work

### 5.1. Conclusions

The study addresses the challenge of detecting falls, characterized by the imbalance between classes and the scarcity of data resulting from difficulties in creating data sets. Using classic ML algorithms (SVM and ANN) and considering datasets UP Fall, UMA Fall, and WEDA Fall, we introduce a new combined dataset that has over 1300 fall samples and 28K ADL samples. The best result of the learning models achieved an average recall of 90.57%, average specificity of 96.91%, and average AUC-ROC of 98.85%, demonstrating the ability to distinguish falls from daily activities. We also study the generalization capabilities of each of the selected datasets. We highlight the specialization of the WEDA Fall set for ADL and the robustness of the UP Fall set in detecting falls. The UMA Fall ensemble presented a balance when generalizing falls and ADL, making it a promising choice for training fall detection models in real-life environments.

When facing the challenge of imbalance between classes, model evaluation should not depend solely on overall accuracy, as this is influenced by the most representative class. Metrics like recall, F1-score and AUC are crucial to provide a more accurate view of performance, especially for the minority class. As for data normalization, its influence varied between datasets and, consequently, across models, emphasizing the importance of adapting normalization according to the specific nature of each dataset and model. The research provides a comprehensive view of model performance in different scenarios, overcoming the limitations of small data sets. These findings provide valuable information for future advances in fall detection, although incorporation of the model into wrist-worn devices was not addressed in this work.

### 5.2. Future Work

For future work, the creation of the data samples should aim to have a sliding window approach during data creation, to have a larger number of data samples. Then, more datasets should be added to this joint dataset in order to increase the variability of fall movements and that more modern learning models be used such as: Convolutional Neural Networks, Recurrent Neural Networks, Decision Trees and Random Forests and Transformers. 

## Figures and Tables

**Table 1:** Some datasets for FDS. The rightmost column shows the number of Activities of Daily Living (ADL) and Falls.

Database	Sensor	Position	ADL/Falls
Smartwatch [30]	Accelerometer	Wrist	2456/107
WEDA Fall [11]	Accelerometer	Wrist	350/619
	Gyroscope		
SisFall [31]	Accelerometer	Waist	2707/1798
	Gyroscope		
TFall [32]	Accelerometer	Thigh	9883/1026
		Handbag	
FARSEEING [33]	Accelerometer	Waist	0/347
	Gyroscope	Thigh	
UP Fall [10]	Accelerometer	Waist	304/255
	Gyroscope	Ankle, Thigh	
		Neck, Wrist	
MobilFall [34]	Accelerometer	Thigh	342/288
	Gyroscope		
	Magnetometer		
DLR [35]	Accelerometer	Waist	961/56
	Gyroscope		
	Magnetometer		
UMA Fall [36]	Accelerometer	Waist, Thigh	322/209
	Gyroscope	Ankle	
	Magnetometer	Chest, Wrist	
FallAllD [37]	Accelerometer	Wrist	-
	Gyroscope	Waist	
	Magnetometer	Neck	

**Table 2:** Types of activities performed in the UP Fall dataset.

1 - Fall forward using your hands	2 - Fall forward using your knees	3 - Fall Back
4 - Fall aside	5 - Falling sitting on the empty chair	6 - Walk
7 - Standing	8 - Sit	9 - Pick up an object
10 - Skip	11 - Lay	

**Table 3:** Types of activities performed in the WEDA Fall dataset.

1 - Walk	2 - Run	3 - Going up and down the stairs
4 - Sit on a chair and stand up	5 - Sitting for a moment, try to get up and fall into a chair	6 - Squat down, tie your shoes and stand up
7 - Tripping while walking	8 - Jump smoothly without falling (trying to reach a high object)	9 - Hitting the table with your hand
10 - Applaud	11 - Open and close door	12 - Falling forward when walking, caused by slipping
13 - Falling to the side when walking, caused by slipping	14 - Falling backwards when walking, caused by slipping	15 - Falling forward when walking, caused by a trip
16 - Falling backwards when trying to sit	17 - Falling forward while sitting, caused by fainting or falling asleep	18 - Falling backwards while sitting, caused by fainting or falling asleep
19 - Falling to one side when sitting, caused by fainting or falling asleep		

**Table 4:** Types of activities performed in the UMA Fall dataset.

1 - Walk	2 - Run	3 - Body flexion (squat)
4 - Skip	5 - Going down stairs	6 - Climbing stairs
7 - Getting in and out of bed	8 - Sitting down and getting up from a chair	9 - Applaud
10 - Raise your hands in the air	11 - Make a call	12 - Open the door
13 - Fall Back	14 - Fall forward	15 - Fall to the Side

**Table 5:** Number of samples from each data source, after running all preprocessing steps. The joint dataset in bold is one of the main results of this paper, which corresponds to joining the three datasets.

	Fall	ADL	Total
UMA Fall	384 (4.93%)	7401 (95.07%)	7785
UP Fall	346 (2.22%)	15,246 (97.78%)	15,592
WEDA Fall	605 (9.27%)	5921 (90.73%)	6526
**Joint dataset**	**1335 (4.46%)**	**28,568 (95.54%)**	**29,903**

**Table 6:** Sensor readings that provide raw data.

Sensor Signals	
Accelerometer: x-axis (g)	Gyroscope: x-axis (rad/s)
Accelerometer: y-axis (g)	Gyroscope: y-axis (rad/s)
Accelerometer: z-axis (g)	Gyroscope: z-axis (rad/s)

**Table 7:** Features extracted from sensor signals.

Maximum amplitude	Average	Root mean square
Minimum amplitude	Median	First Quartile
Number of zero crossings	Skewness	Kurtosis
Third Quartile	Energy	Auto-correlation
Standard Deviation		

**Table 8:** Performance of Base Model and Proposed Model with Datasets UP Fall.

Models		Accuracy	Recall (%)	Specificity (%)
Martinez et al. [10]	RF	95.76 ± 0.18	66.91 ± 1.28	99.59 ± 0.02
	kNN	94.90 ± 0.18	64.28 ± 1.57	99.50 ± 0.02
	ANN	95.48 ± 0.25	69.39 ± 1.47	99.56 ± 0.02
	SVM	93.32 ± 0.23	58.82 ± 1.53	99.32 ± 0.02
Model proposed	ANN	78.29 ± 13.7	94.74 ± 3.14	78.01 ± 13.9
	SVM	94.65 ± 1.60	89.51 ± 4.27	94.76 ± 1.62

**Table 9:** Performance of Base Model and Proposed Model with Datasets Weda Fall.

Models		Accuracy	Recall (%)	Specificity (%)
Marques et al. [11]	3NN	98.05 ± 0.46	98.24 ± 0.36	97.84 ± 0.83
Model proposed	ANN	99.38 ± 2.37	97.03 ± 0.97	97.18 ± 0.81
	SVM	95.10 ± 0.78	88.87 ± 2.64	95.78 ± 0.91

**Table 10:** Performance of Base Model and Proposed Model with Datasets UMA Fall.

Models		AUC (%)	$\max\sqrt{S_{e}S_{p}}$ (%)
Casilari et al [28]	Thresholding	93.5	85.8
Model proposed	ANN	99.8	92.9
	SVM	95.7	89.2

**Table 11:** Model performance combining the datasets UP fall without data normalization.

Dataset	Algorithm	Recall (%)	Specificity (%)	AUC-ROC (%)	F1-Score (%)
UP fall	SVM	89.51 ± 4.27	94.76 ± 1.62	95.86 ± 1.87	40.88 ± 8.99
	ANN	94.74 ± 3.14	78.01 ± 13.9	95.13 ± 1.94	21.35 ± 13.2
UMA fall	SVM	88.40 ± 3.41	95.78 ± 0.91	95.74 ± 1.06	43.19 ± 7.32
	ANN	86.77 ± 6.15	97.18 ± 0.81	99.81 ± 0.08	88.63 ± 3.34
WEDA fall	SVM	88.87 ± 2.64	95.78 ± 0.91	97.18 ± 0.81	77.78 ± 3.71
	ANN	97.03 ± 0.97	97.18 ± 0.8	99.90 ± 0.10	96.80 ± 1.53

**Table 12:** Model performance combining the datasets without and with data normalization.

	Standard		Normalized	
Metrics	SVM	ANN	SVM	ANN
Recall (%)	87.34 ± 1.10	90.57 ± 3.24	89.18 ± 1.48	90.37 ± 2.07
Specificity (%)	93.58 ± 0.71	96.91 ± 1.26	88.25 ± 1.29	91.19 ± 2.58
AUC-ROC (%)	96.14 ± 0.65	98.85 ± 0.28	93.70 ± 0.80	95.67 ± 0.67
F1-Score (%)	52.30 ± 3.00	69.93 ± 6.60	40.82 ± 3.70	48.71 ± 6.38

**Table 13:** Generalization of the model using a dataset for training and the remaining for testing with standardized data.

	UMA Fall		UP Fall		WEDA Fall	
Metrics	SVM	ANN	SVM	ANN	SVM	ANN
Recall (%)	80.126	79.600	89.888	66.632	61.232	68.904
Specificity (%)	91.902	94.411	79.545	86.908	94.118	93.959
AUC-ROC (%)	93.077	93.905	92.071	81.366	91.164	90.688
F1-Score (%)	44.470	52.369	38.626	38.856	35.631	38.677

**Table 14:** Generalization of the model using a dataset for training and the remaining for testing with normalized data.

	UMA Fall		UP Fall		WEDA Fall	
Metrics	SVM	ANN	SVM	ANN	SVM	ANN
Recall (%)	63.751	72.666	90.206	77.735	60.617	61.975
Specificity (%)	93.492	88.032	75.218	81.887	94.608	94.941
AUC-ROC (%)	84.779	89.973	90.984	85.527	87.499	89.498
F1-Score (%)	42.632	34.477	34.712	37.141	38.524	36.299

## Data Availability

The data generation process will be available online.

## Abbreviations

The following abbreviations are used in this manuscript:

MDPI	Multidisciplinary Digital Publishing Institute
ADL	Activity of Daily Living
ARC	Activity Recognition Chain
ANN	Artificial Neural Network
AUC	Area Under the Curve
AUC-ROC	Area Under the Curve ROC
DT	Decision Tree
FDS	Fall Detection System
kNN	K-Nearest Neighbours
ML	Machine Learning
MLP	Multi Layer Perceptron
RF	Random Forest
ROC	Receiver Operating Characteristic
SVM	Support Vector Machines
